# Diffusion of methane in supercritical carbon dioxide across the Widom line

**DOI:** 10.1038/s41598-019-44687-1

**Published:** 2019-06-11

**Authors:** Gabriela Guevara-Carrion, Sergiy Ancherbak, Aliaksandr Mialdun, Jadran Vrabec, Valentina Shevtsova

**Affiliations:** 1 Thermodynamics and Process Engineering, Technical University of Berlin, Ernst-Reuter-Platz 1, 10587 Berlin, Germany; 2 MRC, CP165/62, Université Libre de Bruxelles, Av. F. D. Roosevelt, 50, B-1050 Brussels, Belgium

**Keywords:** Thermodynamics, Chemical physics

## Abstract

Diffusion of methane diluted in supercritical carbon dioxide is studied by experiment and molecular simulation in the temperature range from 292.55 to 332.85 K along the isobars 9.0, 12.5 and 14.7 MPa. Measurements of the Fick diffusion coefficient are carried out with the Taylor dispersion technique. Molecular dynamics simulation and the Green-Kubo formalism are employed to obtain Fick, Maxwell-Stefan and intradiffusion coefficients as well as shear viscosity. The obtained diffusion coefficients are on the order of 10^−8^ m^2^/s. The composition, temperature and density dependence of diffusion is analyzed. The Fick diffusion coefficient of methane in carbon dioxide shows an anomaly in the near-critical region. This behavior can be attributed to the crossing of the so-called Widom line, where the supercritical fluid goes through a transition between liquid-like and gas-like states. Further, several classical equations are tested on their ability to predict this behavior and it is found that equations that explicitly include the density are better suited to predict the sharp variation of the diffusion coefficient near the critical region predicted by molecular simulation.

## Introduction

Carbon dioxide (CO_2_) is ubiquitous, inexpensive and less toxic than most other chemicals that are part of industrial processes. Its critical temperature *T*_*c*_ = 304.13 K is close to that of the ambient and its critical pressure *p*_*c*_ = 7.38 MPa is not too harsh so that its strongly varying density in near-critical states, together with other dependent thermophysical properties, can often be conveniently tuned.

CO_2_ + hydrocarbon systems under high pressure are of particular interest for carbon capture, storage, sequestration, enhanced oil recovery and exploitation of shale gas reserves. Reliable knowledge of transport properties of these systems is necessary for the rational design, control and optimization of the according processes. However, mutual diffusion coefficients are among the least-well studied thermophysical properties of such systems^1^.

CO_2_ captured from energy-conversion processes always contains impurities which may change the properties of CO_2_ significantly. When CO_2_ is stored in depleted reservoirs containing residual hydrocarbons, they mix in states close to its critical point^2,3^. The evaluation of the Péclet number^4^ (ratio of advection to diffusion coefficient) shows that the transport of solute molecules in porous reservoirs is governed by diffusion. Little is known about the effect of impurities in CO_2_ other than water, which motivated this investigation of mutual diffusion of hydrocarbon traces in supercritical CO_2_.

The goal of this work was to study the diffusion behavior of methane (CH_4_) diluted in CO_2_ at high temperature and pressure, particularly in the extended vicinity of the critical point. For this purpose, measurements of the Fick diffusion coefficient were carried out with the Taylor dispersion technique and complemented with molecular dynamics simulations.

Taylor dispersion, also known as peak broadening technique, is widely used for the measurement of the Fick diffusion coefficient. It builds on the diffusive spreading of a small volume of a solution injected into a laminar stream of a carrier fluid. Axial dispersion, mainly resulting from the parabolic flow profile, spreads out the solute pulse longitudinally, while radial diffusion confines the pulse. The combined effects of convective flow and molecular diffusion yield a Gaussian distribution once the mixture has flowed for a sufficient amount of time through a long capillary of uniform diameter. The shape of this distribution at the end of the capillary, known as Taylor peak, is monitored by a detector.

Despite the wide use of this technique for the measurement of diffusion coefficients, its application to supercritical CO_2_ is still under development because its accuracy needs to be improved. To the best of our knowledge, mutual diffusion of CH_4_ infinitely diluted in CO_2_ has not been studied experimentally before. Merely the tracer diffusion coefficient of tritiated methane (CTH_3_) in CO_2_ and in mixtures of CH_4_/CO_2_ has been reported^5^.

For the present experiments, a Taylor apparatus developed in preceding work^6^ was updated. Instead of the usual differential refractometer, a FT-IR spectrophotometer was employed as a detector because it analyzes the internal molecular degrees of freedom that are specific for hydrocarbons. Absorption peaks that correspond to a given molecular vibration mode also exhibit a Gaussian distribution and, in addition, can be analyzed over a wavenumber range. The dispersion coefficient describing such a Gaussian distribution are related to the Fick diffusion coefficient.

Driven by the lack of experimental data on diffusion, substantial efforts have been invested into the development of models for their prediction and estimation. Several predictive equations have been proposed, mainly based on the Stokes-Einstein equation or the rough-hard-sphere model. An overview of the potential and shortcomings these equations can be found in reviews by Suárez *et al*.^7^ and Medina^8^.

On the other hand, molecular simulation has become an alternative for thermodynamic property estimation of CO_2_ and its mixtures^9^. It can readily predict transport property data under supercritical conditions with qualitative and often even with quantitative accuracy to complement experimental data. This field is experiencing a re-growing interest^10–12^ because of the availability of more accurate force fields and faster computers, but also because new experimental data need further interpretation^13^. In fact, molecular simulation can be employed to understand the microscopic interactions and structures that determine the dynamic behavior of supercritical fluids, especially in the vicinity of the critical point and the number of studies combining experiments and molecular simulation is growing rapidly^14–17^. However, the number of molecular simulation studies aiming at transport properties in this region is still small, in part because of the extensive sampling effort required to obtain statistically sound results due to fluctuations in the critical region^13^. A review on the application of molecular modeling and simulation to supercritical fluids was recently published by Stubbs^9^. In particular, Skarmoutsos and Samios^18^ studied the microscopic structure and thermodynamic behavior of the CH_4_/CO_2_ mixture with *x*_CH4_ = 0.2 mol mol^−1^ under supercritical conditions. Feng *et al*.^10^ predicted, among others, the diffusion coefficient of CH_4_ infinitely diluted in CO_2_ at 10.5 MPa for temperatures between 299 and 323 K. In the present work, molecular dynamics simulation was used to interpret and complement present experiments. In particular, self-diffusion coefficients, also termed intradiffusion coefficients in the case of mixtures, Maxwell-Stefan and Fick diffusion coefficients as well as the shear viscosity of the CH_4_/CO_2_ mixture were predicted. The employed force fields for CH_4_ and CO_2_ are rigid, non-polarizable, Lennard-Jones (LJ) based, describing the electrostatics by superimposed point quadrupoles^19,20^.

## Results

Present experiments and molecular simulations sampled mutual diffusion of CH_4_ in supercritical CO_2_ in the extended critical region, but are still somewhat away from the critical point where diffusion anomaly fully occurs according to theoretical predictions^21^, i.e. where the thermodynamic factor and Fick diffusion coefficient tend to zero.

### Thermodynamic factor

Molecular simulations were performed at several compositions near the infinite dilution limit, i.e. *x*_CH4_ = 0.005 to 0.02 mol mol^−1^, in the temperature range between 293.15 and 335 K along the isobars *p* = 9.0, 12.5 and 14.7 MPa. Because equilibrium molecular dynamics was employed, the mixture composition that corresponds to the mutual diffusion scenario was known exactly and the coefficients in the infinite dilution limit were obtained either from extrapolation of the methane intradiffusion coefficient *D*_CH4_ or the Maxwell-Stefan diffusion coefficient *Ð*, since ${D}^{\infty }={\mathrm{lim}}_{{x}_{{\rm{CH}}4}\to 0}{D}_{{\rm{CH}}4}={\mathrm{lim}}_{{x}_{{\rm{CH}}4}\to 0}$ *Ð*. The Fick diffusion coefficient *D* at finite mole fractions was calculated from the Maxwell-Stefan diffusion coefficient, sampled directly from molecular simulations, and the thermodynamic factor Γ, using their relation *D* = Γ*Ð*.

For the calculation of the thermodynamic factor, the composition dependence of the activity coefficients is required, which is usually estimated on the basis of an equation of state or an excess Gibbs energy model fitted to experimental phase equilibrium data. However, the chemical potential sampled with molecular simulation techniques can also be employed to obtain the thermodynamic factor. For this purpose, the chemical potential was calculated with Widom’s test particle method^22^ at two different mole fractions near the target one *x*_*i*_, i.e. *x*_*i*_ ± Δ*x* with Δ*x* = 0.003 mol mol^−1^, and the thermodynamic factor was obtained by numerical derivation.

**Figure 1:**
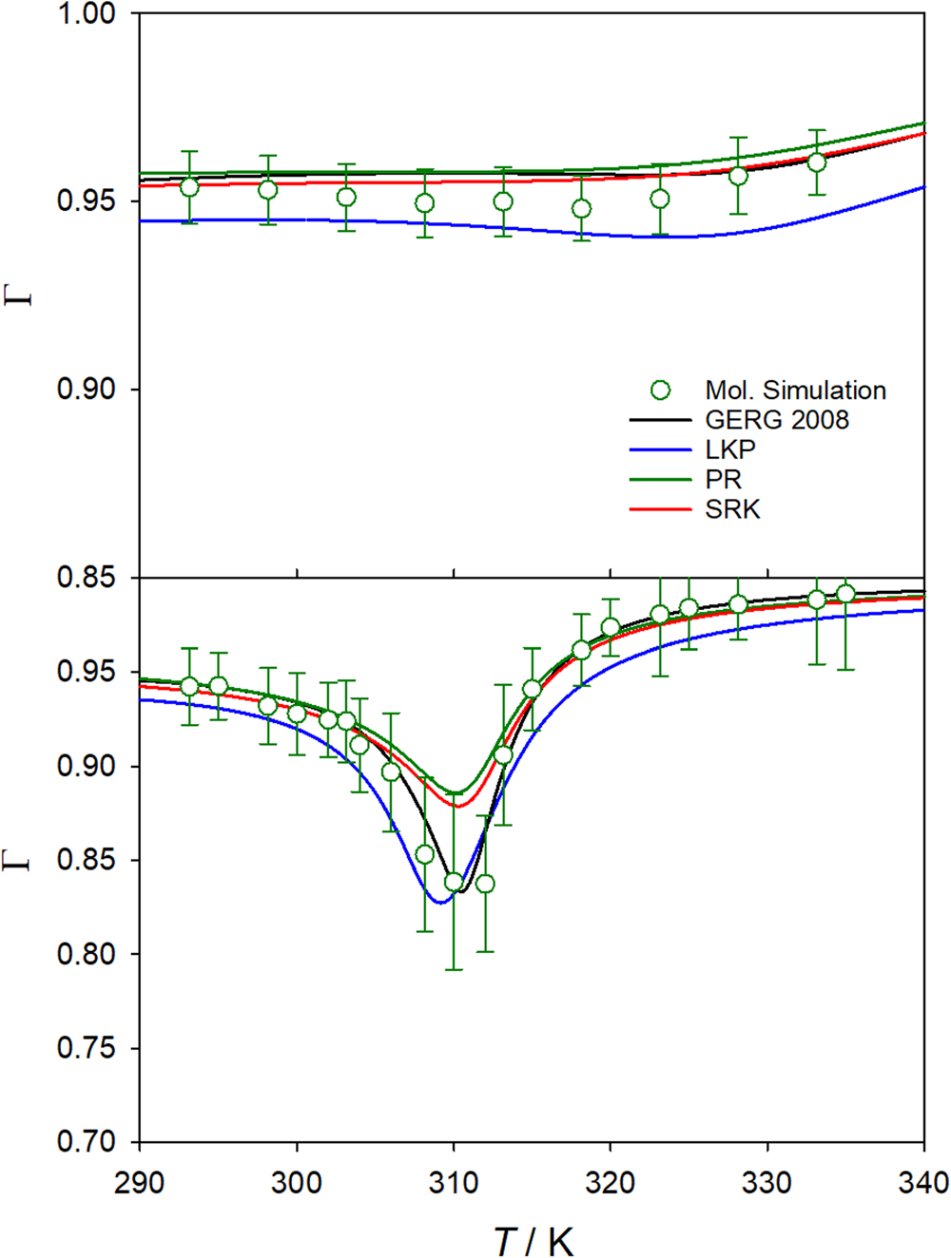
Temperature dependence of the thermodynamic factor of the CH_4_/CO_2_ mixture with *x*_CH4_ = 0.02 mol mol^−1^ at *p* = 9.0 MPa (bottom) and 14.7 MPa (top). Equations of state (lines) are compared with present simulation results (circles).

Furthermore, the thermodynamic factor was also calculated with four different equations of state, i.e. Peng-Robinson^23^ (PR), Lee-Kesler-Plöcker^24^ (LKP), Soave-Redlich-Kwong^25^ (SRK) and GERG-2008^26^. Selected results are shown in Figure 1 for the temperature range from 293.15 to 335 K along the isobars 9.0 and 14.7 MPa, respectively.

It can be seen that all equations of state yield a deep well for the thermodynamic factor along the isobar *p* = 9.0 MPa for temperatures between 300 and 320 K, which is also predicted by molecular simulation. They only slightly differ in magnitude and location of the thermodynamic factor minimum. Low values of the thermodynamic factor can be related to strong density changes due to the proximity to the critical point, where the thermodynamic factor is zero per definition. The good agreement of the molecular simulation results with the GERG-2008 equation of state, which is the most elaborate and accurate correlation available, reinforces both models. Moreover, the well of the thermodynamic factor flattens and approaches unity as CH_4_ concentration decreases, as shown in the Supplementary Material (Figs S7 and S8).

### Composition dependence

**Figure 2:**
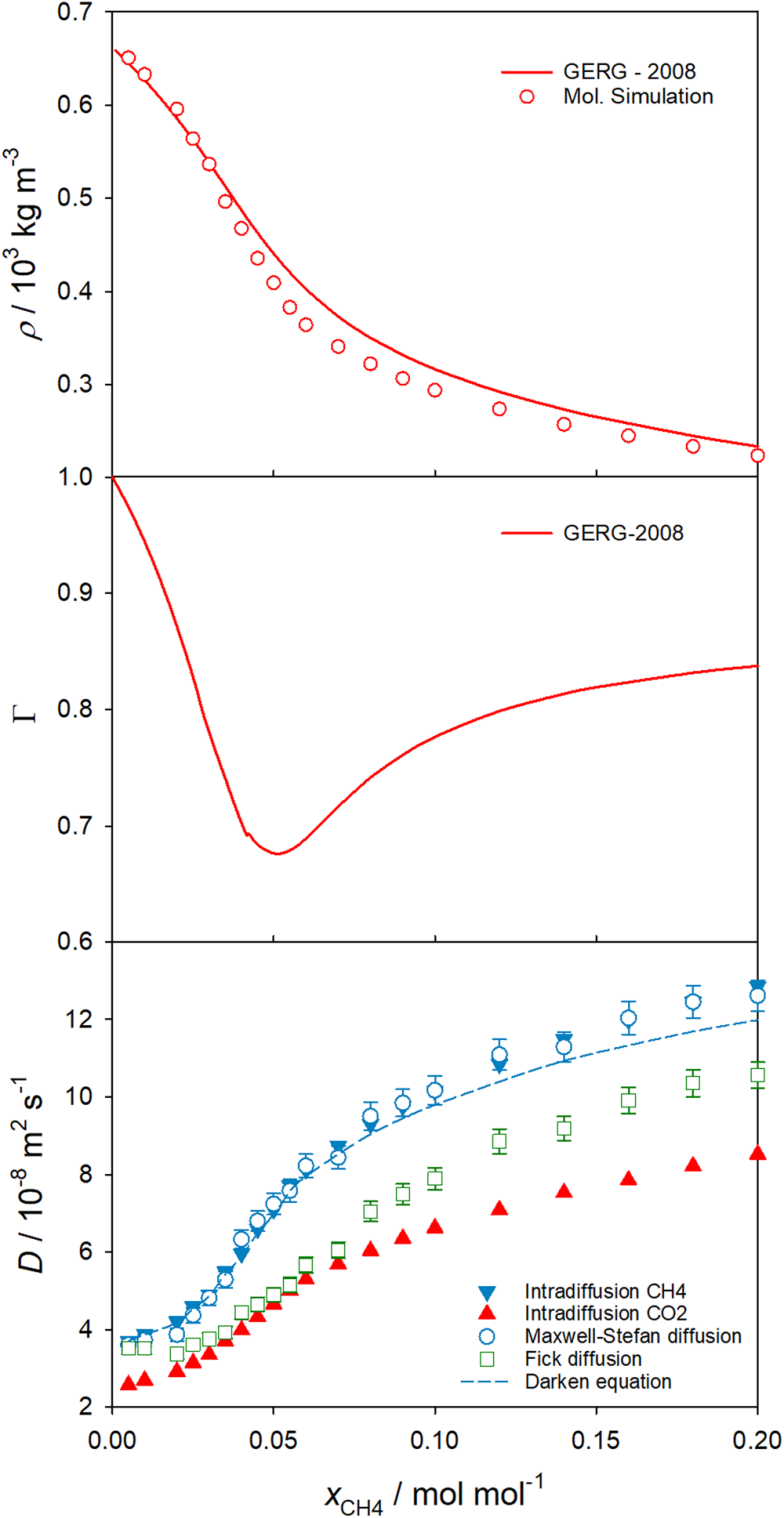
Mole fraction dependence of density (top), thermodynamic factor (center) and diffusion coefficients from molecular simulation (bottom) of the CH_4_/CO_2_ mixture at *T* = 308.15 K and *p* = 9.0 MPa. The solid triangles correspond to the intradiffusion coefficients of CO_2_ (red) and CH_4_ (blue). Present molecular simulation results for Maxwell-Stefan (blue circles) and Fick diffusion coefficients (green squares) are compared with the Maxwell-Stefan diffusion coefficient predicted by the Darken equation (dashed line). The statistical uncertainties of the density and intradiffusion coefficient simulation results are within symbol size.

Per definition, the thermodynamic factor is the unity in the infinite dilution limit so that Fick and Maxwell-Stefan diffusion coefficients attain the same value. For dense liquids, the thermodynamic factor is usually assumed to be unity also near the infinite dilution limit. However, in case of supercritical fluids, the thermodynamic factor may largely differ from unity^12^ for solute mole fractions as low as 0.01 mol mol^−1^. Figure 2 shows a significant mole fraction dependence of the thermodynamic factor of the CH_4_/CO_2_ mixture near the critical point, i.e. at 308.15 K and 9.0 MPa.

As a consequence, the Fick diffusion coefficient may also vary strongly at low solute concentrations, which renders experimental work even more challenging. E.g., at 308.15 K and 9.0 MPa, the Fick diffusion coefficient of the mixture with *x*_CH4_ = 0.05 mol mol^−1^ is approximately 30% higher than that in the extrapolated infinite dilution limit, cf. Fig. 2. It should be noted that the intradiffusion coefficients of CH_4_ and CO_2_ also show a significant composition dependence, which can be explained by the large density variation, cf. Fig. 2.

The considerable injection volume dependence of the Fick diffusion coefficient reported for solutes like acetone^27^, benzene^28^ or naphthalene^29^ in supercritical CO_2_ in the vicinity of its critical point employing the Taylor dispersion technique can be rationalized when Fig. 2 is observed.

### Temperature and pressure dependence

#### Molecular simulation

**Figure 3:**
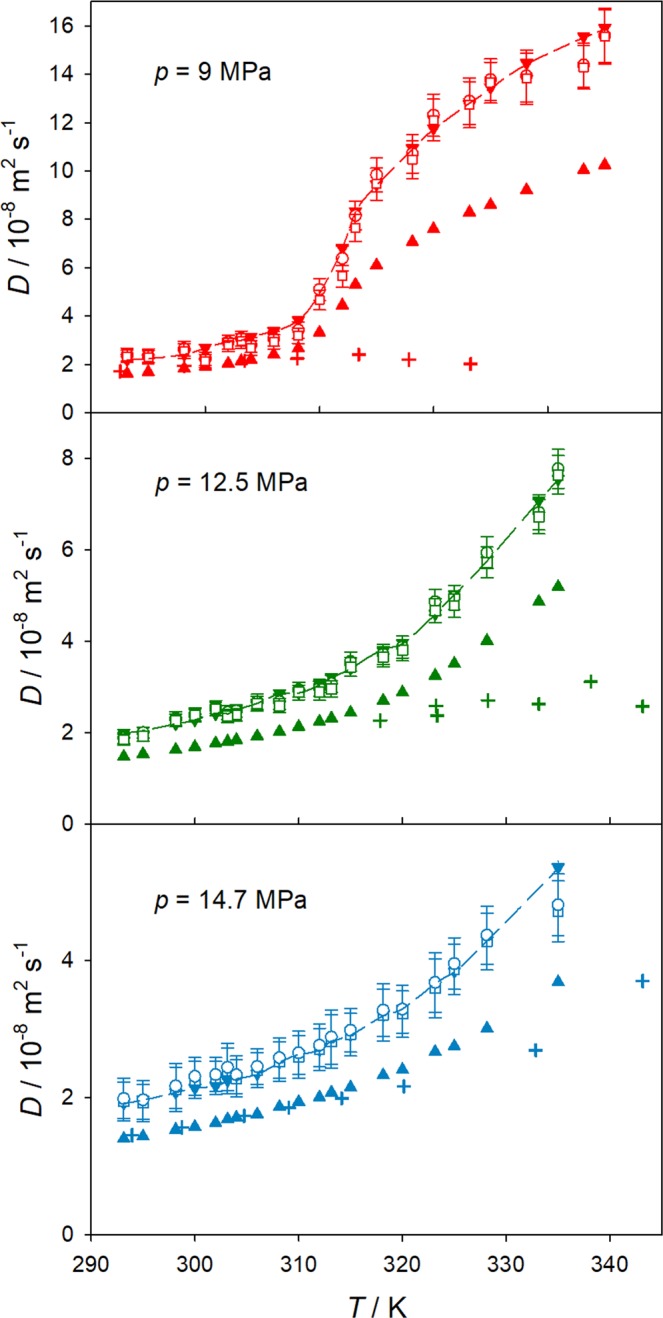
Temperature dependence of intra-, Maxwell-Stefan and Fick diffusion coefficients of the CH_4_/CO_2_ mixture with *x*_CH4_ = 0.01 mol mol^−1^ at *p* = 9.0 MPa (top), 12.5 MPa (center) and 14.7 MPa (bottom). Present simulation results for the intradiffusion coefficient of CO_2_ (triangle up) and CH_4_ (triangle down), Maxwell-Stefan (circles) and Fick diffusion coefficients (squares) are compared with the Maxwell-Stefan diffusion coefficient predicted with the Darken equation (line) and present experimental data (crosses). The statistical uncertainties of the intradiffusion coefficient simulation results are within symbol size.

Figure 3 shows predicted values for the intra- and mutual diffusion coefficients along the isobars *p* = 9.0, 12.5 and 14.7 MPa. In general, the Maxwell-Stefan, Fick and CH_4_ intradiffusion coefficients are equivalent within their statistical uncertainties in the studied temperature range for the mole fraction *x*_CH4_ = 0.01 mol mol^−1^.

Along the lowest isobar *p* = 9.0 MPa and temperatures between 300 and 320 K, a slightly lower value of the Fick diffusion coefficient can be inferred, which is related to the observed deep well of the thermodynamic factor due to the diffusion anomaly near the critical point, cf. Fig. 2. Note that these results do not correspond exactly to the infinite dilution limit, but to a finite mole fraction of *x*_CH4_ = 0.01 mol mol^−1^ that was chosen for the sake of comparison with the present experimental data discussed below. The Darken equation predicts the Maxwell-Stefan diffusion coefficient quite well, suggesting that cross-correlations between unlike molecules do not play a significant role at the studied state points. The intradiffusion coefficient of CO_2_ is lower than that of CH_4_, which is expected because of the larger molecular weight and size of CO_2_. Moreover, the temperature dependence of both intradiffusion coefficients and the self-diffusion coefficient of pure CO_2_ is very similar, which suggests a dominating influence of density. The density dependence of the self-diffusion coefficient of pure CO_2_ in the regarded temperature range is shown in the Supplementary Material (Fig. S4).

**Figure 4:**
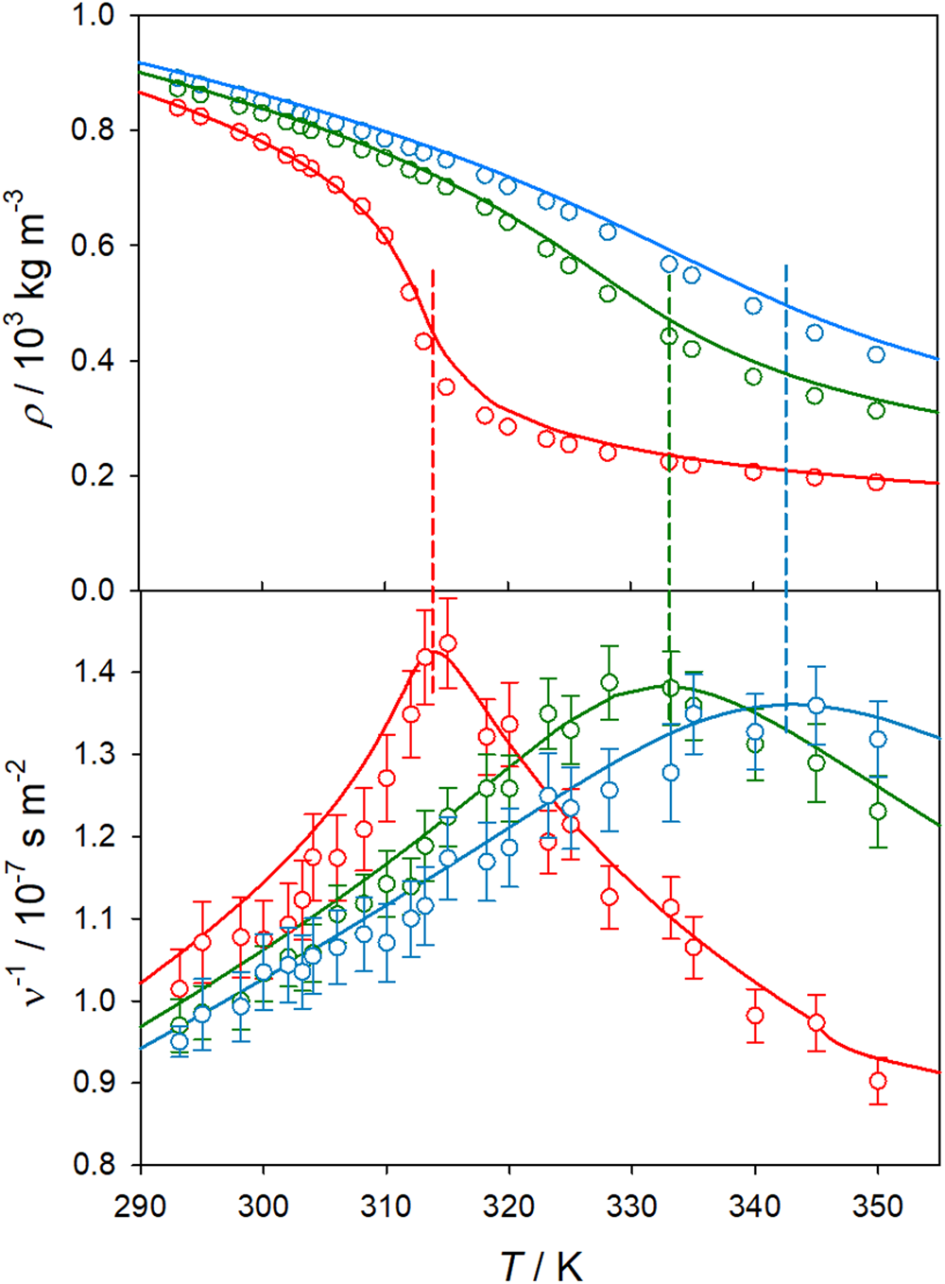
Temperature dependence of density *ρ* (top) and mobility 1/*ν* (bottom) of CO_2_ along the isobars *p* = 9.0 MPa (red), 12.5 MPa (green) and 14.7 MPa (blue). Density from the Span and Wagner equation of state^33^ and the shear viscosity from the Laesecke and Muzny^34^ correlation (lines) are compared with present simulation results (circles). The statistical uncertainties of the density simulation results are within symbol size.

Along the studied isobars, all diffusion coefficients increase with temperature, but in a different manner. Along the highest isobar *p* = 14.7 MPa, all diffusion coefficients increase nearly exponentially with temperature, almost exhibiting Arrhenius behavior. At *p* = 12.5 MPa, the Fick diffusion coefficient shows a change in the slope of the temperature dependence at *T*~320 K, i.e. a curve bend is observed at this temperature, which corresponds to the region where the minimum of the thermodynamic factor was found. This anomalous diffusion bend has also been reported for other supercritical fluids^30^. The temperature dependence of the Fick diffusion coefficient along the *p* = 9.0 MPa isobar, which is closer to the critical point of CO_2_, is far more complex, having a similar shape as the density as a function of temperature, cf. Fig. 4. Here, after an initial quasi-exponential increase with temperature, the Fick diffusion coefficient shows a rapid increase between *T*~308 and 318 K, i.e. the curve shows a drastic change of slope near the Widom line. This behavior corresponds to the transition between liquid-like and gas-like states^31^ and is mainly caused by the free volume increment associated with density variation.

For a better understanding of the diffusion behavior, it should be linked with other thermophysical properties, e.g. density and viscosity, which are important quantities since both depend on temperature and pressure. Consequently, the peculiar behavior of the diffusion coefficients in the proximity of the critical point was related to the evolution of the mobility defined as 1/*ν* = *ρ*/*η*, where *ν* and *η* are the kinematic and shear viscosity, respectively.

**Figure 5:**
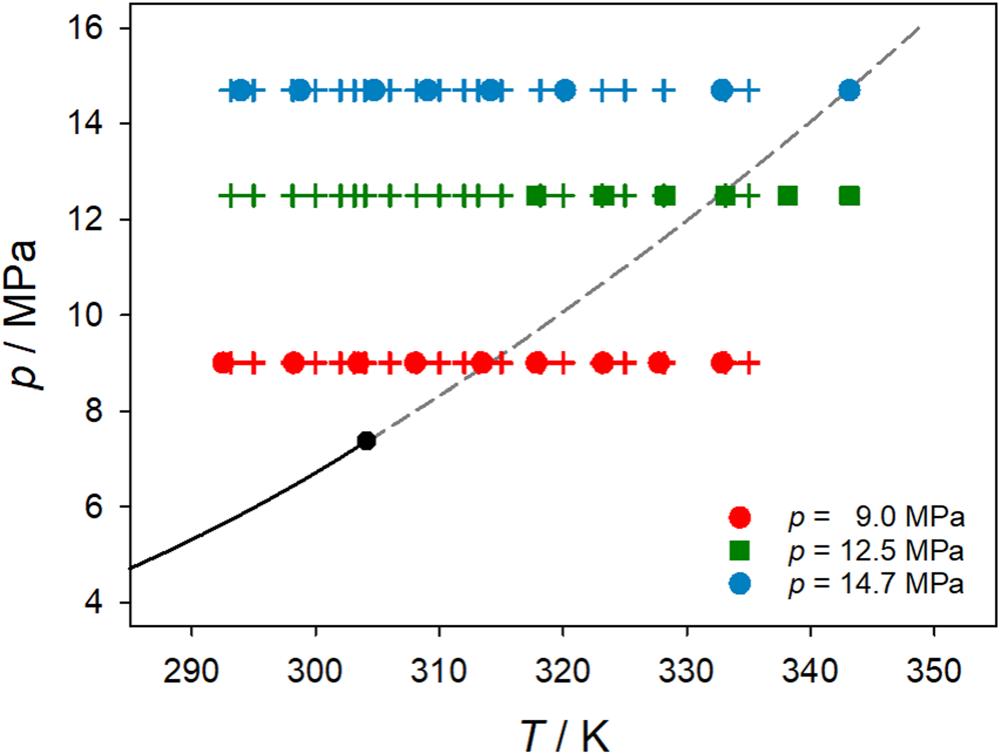
Pressure-temperature phase diagram of pure CO_2_ together with measured (bullets) and simulated (crosses) state points. The vapor pressure curve (solid line) ends at the critical point (bullet) and is extended by the Widom line connecting the maxima of mobility (inverse kinematic viscosity) 1/*ν* (dashed line).

To identify a relation between mutual diffusion at different pressures and the mobility, all measured points were placed into the pressure-temperature phase diagram of pure CO_2_, cf. Fig. 5. A prolongation of the vapor pressure curve into the supercritical region is given by the Widom line, which connects the points of maximum mobility. With increasing pressure, the mobility maximum becomes less pronounced over temperature, cf. Fig. 4. In a narrow band around the Widom line, properties like density, isothermal compressibility, thermal expansion, heat capacities or speed of sound, behave anomalously and exhibit inflection points or extrema, cf. Imre^32^ and references therein. As can be observed in Figure 4, the present molecular model is capable to qualitatively and quantitatively predict the variation of both density and viscosity along the studied isobars. The average deviation of the predicted density and shear viscosity from the Span and Wagner equation of state^33^ and the Laesecke and Muzny^34^ correlation are 3.1% and 3.5%, respectively.

#### Predictive equations

**Figure 6:**
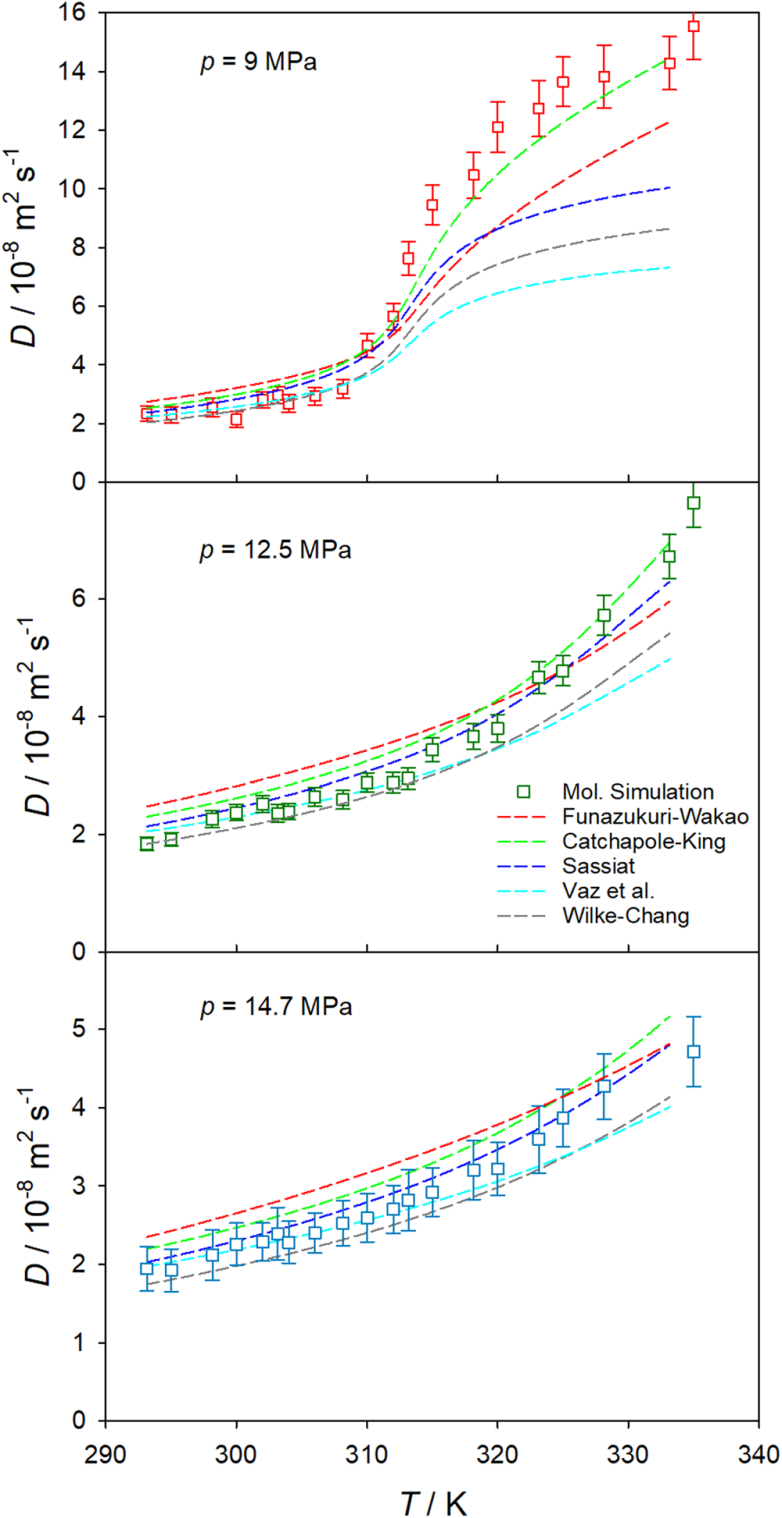
Temperature dependence of the mutual diffusion coefficient at infinite dilution of the CH_4_/CO_2_ mixture along the isobars *p* = 9.0, 12.5 and 14.7 MPa. Molecular simulation results (squares) are compared with selected predictive equations^35,36,40,42,43^ (lines).

The Stokes-Einstein based equations by Wilke-Chang^35^, Sassiat^36^, Tyn-Calus^37^, Reddy-Doraiswamy^38^ and Lai-Tan^39^ as well as the free volume based equations by Catchpole and King^40^, He and Yu^41^, Funazukuri and Wakao^42^ as well as Vaz *et al*.^43^ were considered to assess the mutual diffusion coefficient at infinite dilution predicted by molecular simulation, cf. Fig. 6.

Qualitatively, all predictive equations confirm the present simulation data. The best overall agreement was found for the Sassiat equation with an average deviation of 12%, followed by the Wilke-Chang, Catchpole-King and Vaz *et al*. equations with average deviations of around 13%. Particularly for the higher isobars *p* = 12.5 and 14.7 MPa, the predictions of the Sassiat equation are in excellent qualitative and quantitative agreement with the present simulation results. Along the 9.0 MPa isobar, the Catchpole-King equation shows the best overall agreement with the simulation data, having an average deviation of 15%, whereas the Funazukuri-Wakao equation shows a good qualitative agreement. For this isobar, the Wilke-Chang and Vaz *et al*. equations are in particularly good agreement with present simulation data for temperatures below 315 K, but they fail at higher temperatures or lower density. The rather poor performance of most of the tested predictive equations for the lowest isobar at high temperatures was expected since they were mainly developed for high density fluids far away from the critical region, while Catchapole-King and Funazukuri-Wakao explicitly included the density in their models and consequently perform better in this region.

#### Experiment

**Figure 7:**
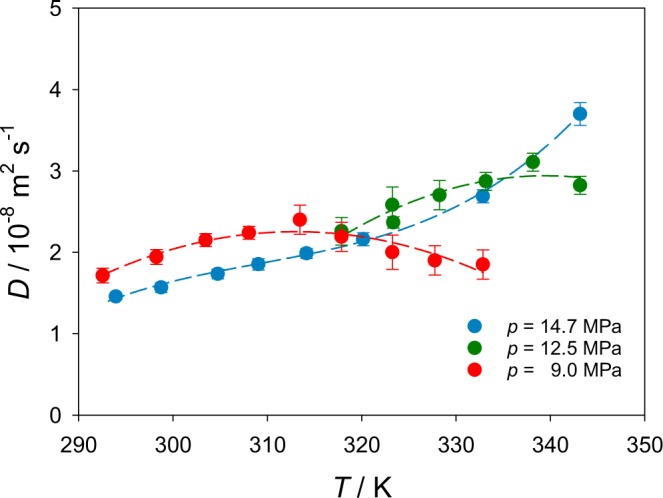
Fick diffusion coefficient of CH_4_ diluted in supercritical CO_2_ measured with Taylor dispersion along the isobars *p* = 9.0, 12.5 and 14.7 MPa as a function of temperature. The dashed lines serve as a guide to the eye.

Present Fick diffusion coefficient data from Taylor dispersion and their standard deviations are given in Table 1. Experimental diffusion coefficient values were averaged over at least five measurements, typically fifteen samples were taken. The reproducibility of the results was generally good and the relative standard deviation varied from 4% to 9% approaching the Widom line. Figure 7 shows the Fick diffusion coefficient as a function of temperature.

**Table 1:** Measured Fick diffusion coefficient *D* and its standard deviation *σ* for CH_4_ in supercritical CO_2_ at different temperatures *T* and pressures *p*.

*p* = 9.0 MPa								
*T*/K	292.55	298.25	303.45	308.05	313.45	317.85	323.25	
*D*/10^−8^ m^2^ s^−1^	1.71	1.94	2.15	2.24	2.40	2.19	2.02	
*σ*/10^−8^ m^2^ s^−1^	0.09	0.09	0.08	0.08	0.18	0.18	0.21	
*p* = 12.5 MPa								
*T*/K	317.85	323.25	323.36	328.25	333.15	338.15	343.15	
*D*/10^−8^ m^2^ s^−1^	2.26	2.58	2.37	2.70	2.62	3.11	2.57	
*σ*/10^−8^ m^2^ s^−1^	0.07	0.11	0.11	0.17	0.22	0.18	0.11	
*p* = 14.7 MPa								
*T*/K	293.95	298.75	304.75	309.05	314.15	320.15	332.85	343.15
*D*/10^−8^ m^2^ s^−1^	1.46	1.57	1.73	1.85	1.99	2.16	2.69	3.70
*σ*/10^−8^ m^2^ s^−1^	0.03	0.06	0.06	0.07	0.06	0.08	0.08	0.14

At *p* = 14.7 MPa, the relation between the Fick diffusion coefficient *D* and temperature is rather simple. *D* increases by more than a factor of two over the considered temperature range. At the lower pressure *p* = 9.0 MPa, the diffusion coefficient shows a clear maximum, cf. Fig. 7. At lower temperatures, the diffusion coefficient increases with temperature, but it starts to decrease at about 313.15 K. Further, the diffusion coefficient at 9.0 MPa is larger than at 14.7 MPa for temperatures <317 K. However, for higher temperatures the trend is just opposite, i.e. values of *D* at *p* = 9.0 MPa are lower than those at *p* = 14.7 MPa.

The measurements at *p* = 12.5 MPa were conducted later in time only to verify the unexpected behavior of diffusion at *p* = 9.0 MPa. The evolution of the diffusion coefficient with temperature at *p* = 12.5 MPa exhibits features that are in between the trends at *p* = 9.0 and 14.7 MPa, cf. Fig. 7. The diffusion curve at *p* = 12.5 MPa has points close to the curve for *p* = 14.7 MPa, however, the last point suggests a decrease of *D* values, which would give it a shape similar to the diffusion behavior at *p* = 9.0 MPa. This finding about the diffusion trends at *p* = 9.0 and 12.5 MPa was not expected in light of previously published results.

Figure 5 shows that measurements at 9.0 MPa cross the Widom line when the temperature approaches 314.15 K, which is precisely the temperature range where a maximum of the Fick diffusion coefficient and an inflection point were observed experimentally and by molecular simulation, respectively. Thus, it is proposed that the existence of an inflection on the diffusion curve is associated with the maximum of mobility, where microscopic fluctuations are particularly strong. The isobar *p* = 12.5 MPa also intersects with the Widom line and is influenced by anomalies associated with this region, but less pronounced than at *p* = 9.0 MPa. Since density variations upon temperature increase are much sharper at lower pressures, where the system is closer to the critical point of CO_2_ (cf. Fig. 4), there is a stronger impact on the peak of mobility and diffusion values. They become more pronounced at lower pressures closer to the critical point. Thus, the potential effect on diffusion related to the crossing of the Widom line at *p* = 12.5 MPa is weaker than at *p* = 9.0 MPa.

This also explains the monotonic increase of the diffusion coefficient with temperature at *p* = 14.7 MPa, where all measurements were carried out far away from the critical point and only the last state points approach the Widom line. Furthermore, even when crossing the Widom line at this pressure, the effect on diffusion is weaker, as the temperature dependence of density is smoother and hence its influence on diffusion is dampened.

**Figure 8:**
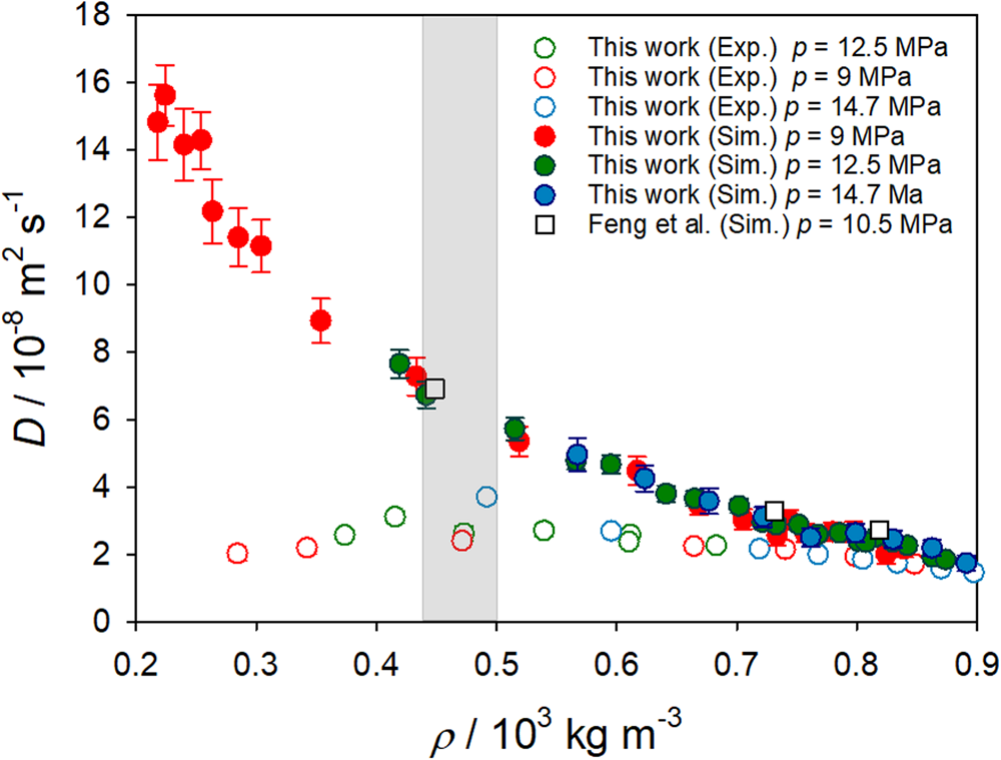
Solvent density dependence of the Fick diffusion coefficient for the CH_4_/CO_2_ mixture with *x*_CH4_ = 0.01 mol mol^−1^ along the isobars *p* = 9.0, 12.5 and 14.7 MPa. Present simulation results are compared with simulation results by Feng *et al*.^10^ at 10.5 MPa and present experimental data. The shaded region indicates the density range of the Widom line. The uncertainties of the experimental results are within symbol size.

When present measurements are compared to present simulation results, there is a good qualitative agreement along the highest isobar, cf. Fig. 3. Because of the underestimation of CO_2_ density (cf. Fig. 4) and shear viscosity (cf. Supplementary Material Fig. S6) in the studied temperature and pressure ranges, predicted diffusion coefficients might be expected to be somewhat higher than experimental values. Quantitatively, experimental data are similar to the predicted intradiffusion coefficient of CO_2_ in the mixture, being therefore lower than the Fick diffusion coefficient predicted by simulation. A similar agreement can be found for the lowest isobar *p* = 9.0 MPa, but only for temperatures below 310 K. At higher temperatures, the experimental data show an unusual behavior, which was not confirmed by molecular simulation. Contrary, the Fick diffusion coefficient continues to increase with increasing temperature and decreasing density. Figure 8 shows the Fick diffusion coefficient in the infinite dilution limit as a function of CO_2_ density. It can be seen that the curves for the studied isobars are comparable, reinforcing the observed substantial influence of density on diffusion. This strong density dependence was also observed by molecular simulation for the self-diffusion coefficient of pure CO_2_ at the studied state points, cf. Supplementary Material (Fig. S3).

The approach to the critical point or Widom line that is associated with large density variations affects molecular simulation and Taylor dispersion techniques negatively. In fact, the larger uncertainties of the simulation data are expected at *p* = 9 MPa and temperatures between 310 and 320 K, where a maximum underestimation of 13% for the predicted CO_2_ density and shear viscosity are given. Further, simulation results show more scatter and larger statistical uncertainties in this region. Experimentally, the largest errors for the measured state points are observed in the vicinity of the Widom line, which do not exceed 11%. The presence of a maximum of the Fick diffusion coefficient of an infinitely diluted solute dissolved in supercritical CO_2_ has been reported in the literature before^28^, but was challenged by other experimental work^44,45^ and the injection volume was identified as one of the factors which affects the density in this region. Further, measurements near the critical point have been commonly associated with the presence of peak tailing^4,45–47^. Indeed, the measurements at *p* = 9.0 MPa exhibit more scatter than at *p* = 14.7 MPa, especially in the low density (high temperature) region. To visualize these uncertainties, a symmetric “mirror image” of the dispersion peak relative to its maximum was generated for all asymmetric peaks. The idealized peaks were then employed to calculate the diffusion coefficient. A difference between the diffusion values obtained from the dispersion peaks and their mirror images was significant only near the Widom line, i.e. *T* > 310 K. Nevertheless, the original and the mirrored peaks of the Gaussian distribution yield diffusion values that show an inflection on the trend-curves of *D* as a function of temperature and/or density.

Additional confidence in the present results with respect to the presence of a local maximum on the diffusion curve *D*(*T*) was provided by a previous study on supercritical CO_2_ ^48^, where an increase in the diffusion flux was observed while conducting experiments of CO_2_ permeation through elastomers. It was evidenced that the maximum of the diffusion flux of CO_2_ corresponds to the thermodynamic state with a mobility maximum, which is located on the Widom line.

Some speculations can be found in the literature about a minimum^27,28,49–51^ or slowing down^45,52–54^ of the Fick diffusion coefficient of different compounds, such as naphthalene, benzene or acetone, when the critical point of the solvent is approached. From the other side, theoretical estimations based on irreversible thermodynamics and the thermodynamic factor, e.g. by Clifford and Coleby^55^ for naphthalene in supercritical CO_2_, indicate that the infinite dilution mutual diffusion coefficient should not decrease approaching the critical point. It should be noticed that present and other^12^ simulation works support the presence of a slowing down of the Fick diffusion coefficient near the critical region for a finite solute concentration that becomes less pronounced as the infinite dilution limit is approached, i.e. when the thermodynamic factor becomes unity. The question still remains open: What is the physical nature of the observed slowing down of diffusion for *ρ* < *ρ*_*c*_ with the Taylor dispersion technique and why it is not observed by molecular simulation?

## Discussion

A comprehensive study on diffusion coefficients of CH_4_ diluted in supercritical CO_2_ was conducted in the temperature range from 292.55 K to 332.85 K for the isobars 9.0, 12.5 and 14.7 MPa. Complementary approaches, i.e. experiment, molecular simulation and predictive equations, were considered to gain an understanding of the diffusion behavior of this mixture.

Gaseous CH_4_/CO_2_ mixture with a mole fraction of *x*_CH4_ = 0.2052 mol mol^−1^ was injected into a pure supercritical CO_2_ flow and the Fick diffusion coefficient was measured with the Taylor dispersion technique. On the whole, the reproducibility of the experimental results was good in terms of Taylor peak shape and diffusion coefficients.

The Maxwell-Stefan diffusion coefficient was sampled with equilibrium molecular dynamics, employing rigid, non-polarizable force fields based on LJ sites and superimposed point quadrupoles, employing the Green-Kubo formalism. The thermodynamic factor was calculated on the basis of chemical potential data sampled with Widom’s test particle method. In this way, the Fick diffusion coefficient was determined by molecular simulation consistently on the basis of the chosen force field.

Analyzing diffusion of the supercritical CH_4_/CO_2_ mixture, present results underline the importance of the Widom line, where several thermophysical properties (e.g. density, compressibility, thermal expansion) become very sensitive to temperature and pressure variations and can therefore change drastically along supercritical paths.

A comparison between predictive molecular simulation data and experimental results for the Fick diffusion coefficient was performed in the light of the density dependence. We examined this comparison from the perspective of proximity to the Widom line, i.e. the values of the Fick diffusion coefficient at various densities as compared to its values near the Widom line. A favorable agreement was observed for higher mixture densities. In the density range along the Widom line, the experimental results display a local maximum of the Fick diffusion coefficient with a subsequent decrease towards lower densities, while the simulation results indicate an increase. Qualitatively, all predictive equations support these numerical findings. Possible reasons for these diverging behaviors were thoroughly discussed.

Molecular simulation and a range of equations of state disclosed a strong composition dependence of the thermodynamic factor approaching infinite dilution, which may lead to a significant composition dependence of the diffusion coefficient. On the experimental side, the concentration of the solute in the experimental cell depends on the thermodynamic pressure-temperature path after injection. Clearly, diffusion behavior in the vicinity of Widom line and the critical point deserves future experimental and theoretical investigations.

## Methods

### Molecular simulation

In this work, equilibrium molecular dynamics simulations of CH_4_ diluted in supercritical CO_2_ were performed, employing rigid, non-polarizable force fields based on LJ sites and superimposed point quadrupoles^19,20^. The capability of the chosen CO_2_ force field to adequately predict self-diffusion coefficient, shear viscosity and thermal conductivity of the pure fluid over a wide range of thermodynamic conditions has been reported in previous work^20^. Also for pure CH_4_, an excellent agreement was found between the predicted self-diffusion coefficient and available experimental data in the studied temperature and pressure range. A graphical comparison can be found in the Supplementary Material (Fig. S5).

Transport coefficients were sampled with equilibrium molecular dynamics and the Green-Kubo formalism. This formalism was preferred over non-equilibrium methods because intra- and mutual diffusion coefficients as well as shear viscosity can be sampled concurrently. The general Green-Kubo expression for an arbitrary transport coefficient Ξ is given by

1 $${\rm{\Xi }}=\frac{1}{G}{\int }_{0}^{\infty }\,dt\langle \dot{{\bf{A}}}(t)\cdot \dot{{\bf{A}}}(0)\rangle ,$$

where *G* is a transport property specific pre-factor, **A** the related perturbation, $\dot{{\bf{A}}}$ its time derivative and the brackets 〈…〉 denote the ensemble average. The working equations for the determination of the different transport properties have been published e.g. in ref.^56^ and are not repeated here. All simulations were carried out with the program *ms*2^57^ and the technical details are given in the Supplementary Material.

### Experiment

#### Apparatus

The Taylor dispersion apparatus used in this work consisted of four modules: a carrier fluid conditioning device, the CO_2_ delivery system with a solute injection valve, the air bath thermostat housing the diffusion capillary and a FT-IR detector. A detailed description of the apparatus can be found elsewhere^6^, here only the last module is briefly described.

The Taylor peak was monitored at the outlet of the dispersion tube by a FT-IR spectrophotometer (Jasco FT-IR 4100) with an accuracy of ±0.01 cm^−1^ and a resolution of 4 cm^−1^ that was equipped with a high pressure demountable cell (Harrick). Most refractive index detectors (RID) operate at low pressure <0.5 MPa so that restriction tubes must be introduced before RID^4,58^ when they are connected to a high pressure diffusion column, while FT-IR detectors can be equipped with a high pressure cell. To avoid the associated perturbations, an FT-IR detector was selected here to study diffusion. The FT-IR detector worked at high pressure and the flow was decompressed after passing the detector. Its optical windows were made of ZnSe, allowing for a maximum working pressure of 25 MPa. The thickness of the cell was 150 *μ*m. Using spare windows from sapphire would have allowed to increase the working pressure even up to 50 MPa. Another advantage of FT-IR detectors is their ability to carry out measurements for a broad range of wavenumbers simultaneously. The detector was connected to a computer for digital data acquisition using the Spectra Manager by Jasco.

Response curves, i.e. the variation of the solute concentration with time, were monitored by means of absorbance spectra at wavenumbers corresponding to different molecular vibration modes. The procedure to select the working wavenumbers, the experimental protocol and the fitting procedure can be found in the Supplementary Material.

#### Materials

CO_2_ with a claimed purity of 0.99998 mol mol^−1^ was purchased from Air Liquide in a bottle in its vapor-liquid equilibrium state, i.e. with a nominal pressure *p* = 6.4 MPa at *T* = 298 K. The gaseous CH_4_/CO_2_ mixture with *x*_CH4_= 0.2052 mol mol^−1^ was also prepared and supplied by Air Liquide at *T* = 288.15 K and *p* = 3.3 MPa.

#### Evolution of the solute mixture after injection

The operation of a Taylor set-up with a carrier flow and an injection at high pressure is challenging because pressure equilibrium has to be maintained. Pressure mismatch can lead to problems, depending on the pressure ratio between carrier fluid and injection loop.

Several questions had to be answered for the CH_4_/CO_2_ mixture: Does it experience a phase transition, how does its density change upon injection and what is the composition of solute in the high pressure cell? The carrier fluid was throughout pure CO_2_ at *T*_*exp*_ = 292.55 to 332.85 K and *p*_*exp*_ = 9.0 to 14.7 MPa. Its temperature was kept constant by the regulated thermal air bath, while pressure constancy was ensured by a high pressure pump and a back-pressure regulator. The injected sample was the gaseous CH_4_/CO_2_ mixture with a mole fraction *x*_CH4_ = 0.2052 mol mol^−1^ which was stored in a bottle at ambient temperature *T*_*A*_ = 288.15 K and a pressure of *p*_*inj*_ = 3.3 MPa. Its density *ρ* = 63.5 kg m^−3^ was estimated with the GERG-2008^26^ equation of state, which is the standard reference of the REFPROP 10.0 database^59^ maintained by the National Institute of Standards and Technology.

**Figure 9:**
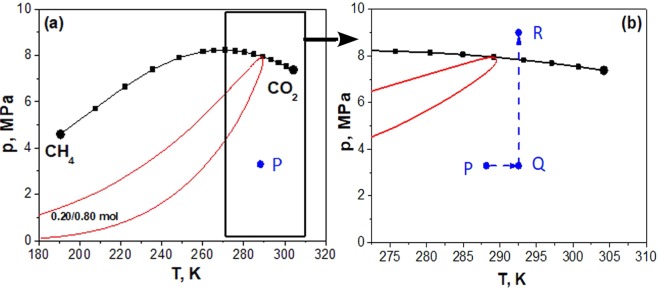
Thermodynamic path of the CH_4_/CO_2_ mixture. (**a**) The critical points^60^ of pure CH_4_ and CO_2_ are indicated by black bullets. The spacing between the black squares along the critical line corresponds to mole fraction increments of Δ*x* = 0.05 mol mol^−1^. The red curve is the vapor-liquid coexistence envelope of the mixture with *x*_CH4_ = 0.20 mol mol^−1^. (**b**) Magnified view of panel (a) depicting the thermodynamic path during the experiment. Point P corresponds to the initial state (storage bottle at ambient temperature *T*_*A*_ ≥ 288 K), point Q corresponds to the state inside the injection loop and point R corresponds to the state of the sample in the carrier flow.

The mixture was supplied directly from the bottle into the injection valve with a nominal calibrated volume of *V*_0_ = 20 *μ*L to achieve a reasonable signal-to-noise ratio. Since the injection valve was located inside the thermal air bath, the mixture adopted the target temperature *T*_*exp*_ in the injection loop. It is reasonable to presume that temperature equilibration *T*_*A*_ → *T*_*exp*_ occurred rapidly and that the mixture remained at *T*_*exp*_ and *p*_*inj*_ before being injected into the carrier flow. Considering the lowest temperature-pressure point studied here (*T*_*exp*_ = 292.55 K, *p*_*exp*_ = 9.0 MPa) the density of the mixture under these conditions was *ρ* = 362.03 kg m^−3^ according to ref.^26^. In the next step, the mixture was injected into the carrier flow, where it experienced a fast transition *p*_*inj*_ → *p*_*exp*_ at constant temperature *T*_*exp*_. The critical line and the vapor-liquid coexistence envelope of the CH_4_/CO_2_ mixture are shown in Figure 9a.

To explore whether the mixture experienced a phase transition upon injection, its thermodynamic path is presented in a pressure-temperature diagram in Fig. 9b. The most interesting transition occurred between points Q and R. It can be seen that the path between these state points is rather far away from the phase envelope, which corresponds to the mixture composition at point Q. Only after reaching point R, the composition of the injected mixture may have started to change. This change in composition will shift the phase envelope further to the right side of the diagram. However, this shift of the envelope will only occur when mixture already reached point R. Thus, it may safely be assumed that the mixture did not experience phase change.

**Figure 10:**
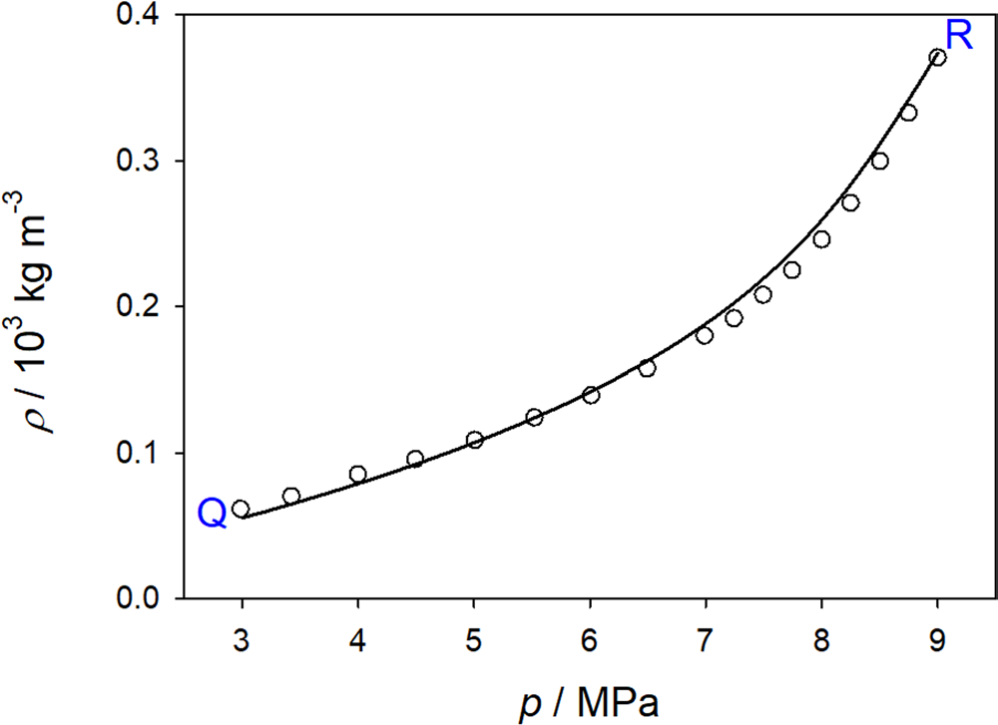
Density dependence of the CH_4_/CO_2_ mixture with *x*_CH4_ = 0.2 mol mol^−1^ upon pressure at 292.55 K along the isothermal path Q-R shown in Figure 9b. The GERG-2008 equation of state^26^ (line) is compared with present simulation results (circles).

The variation of the mixture density along path Q–R as shown in Figure 9b is depicted in Fig. 10. It may be concluded from the density behavior that the compression *p*_*inj*_ → *p*_*exp*_ was a smooth transition from a gaseous to a supercritical state. The density of the mixture *ρ*_*inj*_ = 61.76 kg m^−3^ at *p*_*inj*_ = 3.3 MPa changed to *ρ*_*exp*_ = 362.03 kg m^−3^ at *p*_*exp*_ = 9.0 MPa. From the mass balance *m* = *ρ*_*inj*_ *V*_0_ = *ρ*_*exp*_V*, it follows V* = *V*_0_*ρ*_*inj*_/*ρ*_*exp*_ ≈ 0.17 *V*_0_. The injection volume after compression V* has to be used for the estimation of the concentration of the pulse. The estimation based on the peak shape of the injected sample for *p* = 9.0 MPa and *T* = 313 K provides *x*_*exp*_ = 0.0156 mol mol^−1^ instead of *x*_CH4_ = 0.2052 mol mol^−1^. It should be noted that this estimate is valid for the given thermodynamic path and had to be re-estimated for each sampled temperature-pressure point. In fact, such a noticeable decrease of the injected sample volume should necessarily cause a significant disturbance. Practically, the initial concentration of CH_4_ would hardly create a step-wise profile bounded by volume V*, but would rather resemble a Gaussian covering the entire initial volume *V*_0_.

## Supplementary information

Supplementary Information File

## Data Availability

All data analysed during this study are included in this published article and its Supplementary Information files. Other generated data during the current study are available from the corresponding author upon request.
