# Supplementary material for: Diffusion of methane in supercritical carbon dioxide across the Widom line
Source: Sci Rep. 2019 Jun 11;9:8466. doi: 10.1038/s41598-019-44687-1 (PMC6560060; doi:10.1038/s41598-019-44687-1)
Supplement: Supplementary file 1 — Supplementary Information File [file 41598_2019_44687_MOESM1_ESM.pdf]

Supplementary material to:

Diffusion of methane in supercritical carbon dioxide across the Widom line

Gabriela Guevara-Carrion,[1] Sergiy Ancherbak,[2] Aliaksandr Mialdun,[2] Jadran Vrabec,[1, a)] and Valentina Shevtsova[2]

[1)] *Thermodynamics and Process Engineering, Technical University Berlin, Ernst-Reuter-Platz 1, 10587 Berlin, Germany*

[2)] *MRC, CP165/62, Université Libre de Bruxelles, Av. F.D. Roosevelt, 50, B-1050, Brussels, Belgium*

[a)] Electronic mail: vrabec@tu-berlin.de

EXPERIMENTAL DETAILS

A. Selection of working wavenumbers

Prior to the experiments, the transmittance of the IR spectra in pure supercritical CO_2 and the CH_4/CO_2 mixture was measured. The presence of the involved molecular species in the flow of supercritical CO_2 can easily be detected with an FT-IR spectrophotometer. From the spectra for pure supercritical CO_2, the two wavenumber regions with the highest transmittance of IR radiation were determined, i.e. 800 to 2200 cm^{-1} and 2500 to 3500 cm^{-1} (Fig. S1a). In turn, from the IR spectra of the CH_4/CO_2 mixture, wavenumbers were selected where absorption of IR light is maximal, i.e. transmittance is minimal (Fig. S1b).

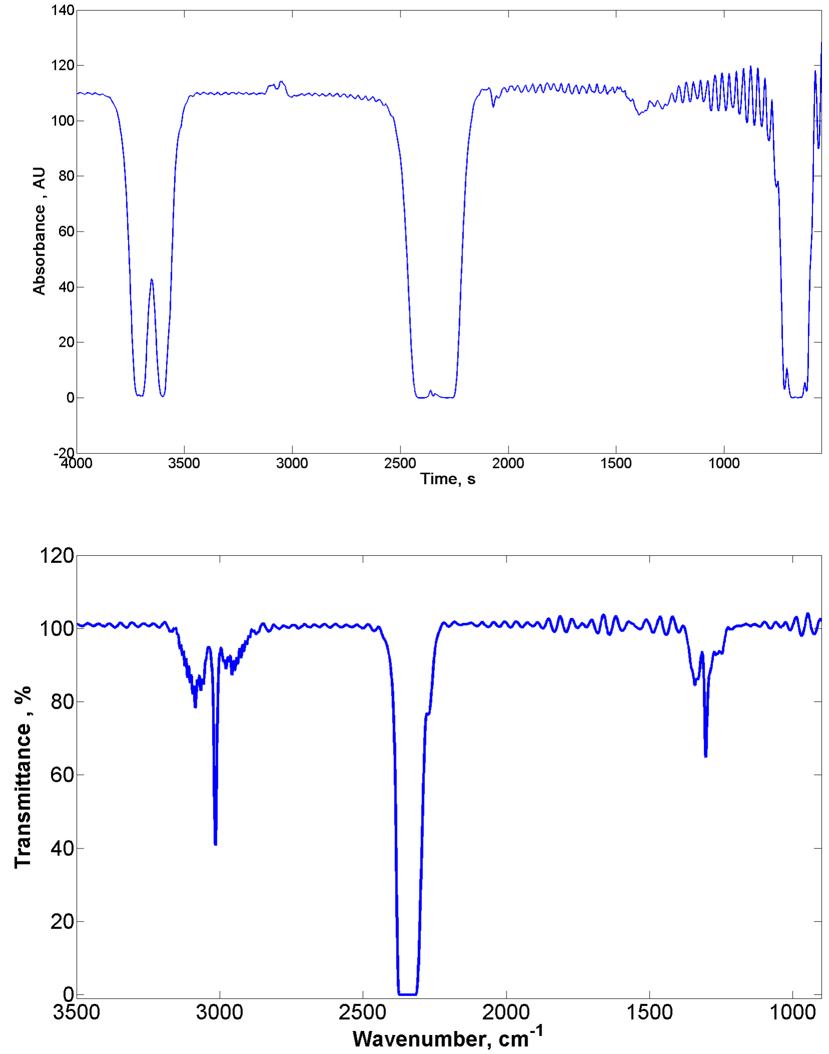

FIG. S1. Transmittance in the IR spectra for (a) pure supercritical CO_2 at $T = 320.15\,\mathrm{K}$ and $p = 14.7\,\mathrm{MPa}$, (b) CH_4/CO_2 mixture with $x_{\mathrm{CH4}} = 0.2052$ mol mol^{-1} at ambient temperature and $p = 3.3\,\mathrm{MPa}$.

Signal absorption of was monitored at wavenumbers 1303.6 and 3015.2 cm^{-1}. These wavenumbers correspond to C-H bending and stretching modes of CH_4 molecules, respectively, and are in good agreement with literature data[1]: 1306.2 and 3018.9 cm^{-1}. Moreover, for supercritical CO_2, these vibrational modes appear in wavenumber regions where the highest transmittance of IR light is observed and all detected signals can be related to the presence of CH_4. In addition, around the wavenumber 2000 cm^{-1}, the absorption of IR light in both CO_2 and CH_4 is minimal (transmittance maximum), which is useful to control the baseline during experiments.

Furthermore, the dependence on the path length in the high pressure cell was also examined. For this purpose, measurements (not very close to the Widom line) were performed using spacers with varying thickness: 25 μm, 150 μm and 500 μm. The results showed that thinner spacers yield more symmetric peaks but have a smaller amplitude, decreasing the signal-to-noise ratio, that adversly affects the estimation of the diffusion coefficient. Thicker spacers led to a good signal-to-noise ratio but showed the largest peak asymmetry. Thus, the optimal spacer thickness was chosen to be 150 μm, being a good compromize for signal-to-noise and peak symmetry.

B. Experimental protocol

A typical experiment was carried out as follows: First, the temperature of both thermostats was set to the desired values. The cryothermostat always operated at 269.15 K to ensure that CO_2 entered the pump in the liquid state. The second thermostat was set to the experimental target temperature. Both thermostats worked for at least two hours before measurements were started to attain temperature stabilization. The pressure was controlled by a back pressure regulator and the pump was set to the highest possible flow rate (4.0 mL min^{-1}). When the pressure approached the desired value, the flow rate was decreased step-wise to its target value, which was a function of the carrier fluid viscosity. The carrier fluid (pure CO_2) flowed through the dispersion tube for at least one hour to ensure a stationary baseline. Once steady-state flow and thermal equilibrium were attained, measurements were initiated. A 20 μL pulse of the CH_4/CO_2 mixture was then injected into the carrier flow. Each measurement lasted for about 1.5 to 3 hours. To avoid peak overlaps and other perturbations, only one injection was loaded per run. The measurement for each pair of temperature and pressure was repeated at least five times.

It is known that the secondary flow[2–6] generated by the centrifugal force can significantly influence the results. However, the error caused by this effect is negligibly small when $\mathrm{DeSc}^{1/2} < 8$, where $\mathrm{De} = 2\sqrt{R_0/R_c}R_0\,\bar{u}/\nu$ and $\mathrm{Sc} = \nu/D$ are the Dean and the Schmidt numbers, respectively[4]; $\bar{u}$ is the average velocity and ν the kinematic viscosity of the carrier flow. To avoid secondary flow, all measurements were carried out at low flow velocities with $\mathrm{DeSc}^{1/2} < 8$.

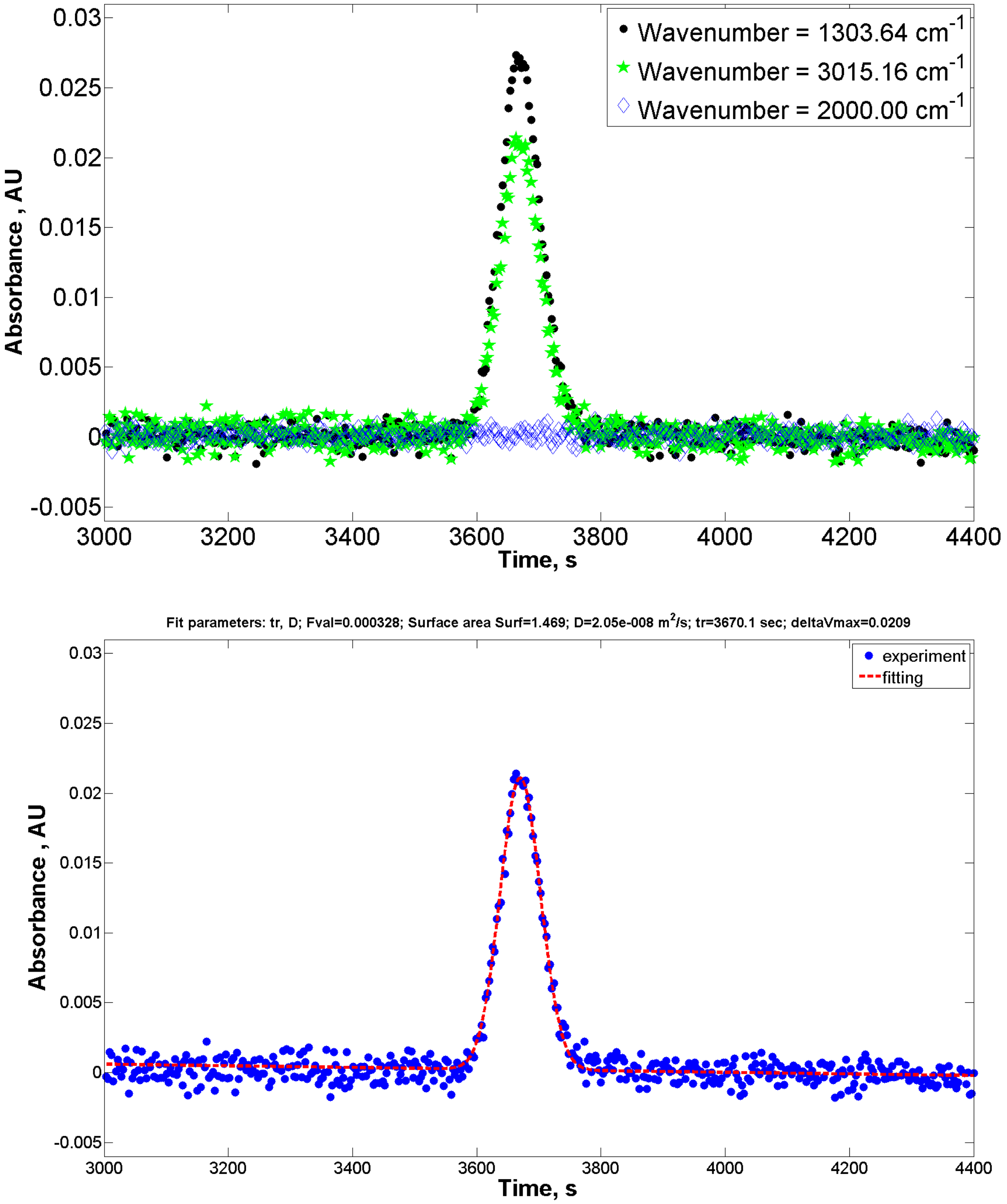

FIG. S2. (a) Experimental absorption signals at different wavenumbers of the CH_4/CO_2 mixture in supercritical CO_2 at $T = 320.15\,\mathrm{K}$ and $p = 14.7\,\mathrm{MPa}$. (b) Experimental absorption curve obtained at $1303.64\,\mathrm{cm}^{-1}$ (bullets) and its correlation with Eq. (1) (line).

C. Taylor peak shape analysis

Exemplarily, experimental signals at different wavenumbers for the CH_4/CO_2 mixture in supercritical CO_2 are shown in Fig. S2a. The two peaks correspond to the wavenumbers 1303.6 and $3015.2\,\mathrm{cm}^{-1}$ at which the absorbance of the signals is maximal and the third

signal was recorded at the wavenumber 2000.0 cm^{-1}, where the absorbance is minimal. This signal was stable and did not show any unusual variations during the measurements so that it was used as a baseline.

As discussed in the literature[4,6,7], the Fick diffusion coefficient can be calculated from the variation of concentration over time t. The employed detector did not sample concentration directly, but the absorbance of the solute. According to the Beer-Lambert law, it can be assumed that small changes in concentration are proportional to variation of absorbance. Then, the absorption of the solute (in absorption units) averaged over the cross section at the end of the diffusion tube is

$$A(t) = A_0 + A_1 t + A_2 t^2 + \Delta A \sqrt{\frac{t_R}{t}} \exp\left(-\frac{12\, D\, (t - t_R)^2}{R_0^2\, t}\right), \qquad (1)$$

where the three first terms $A_0 + A_1 t + A_2 t^2$ consider the drift and curvature of the baseline due to small concentration and temperature variations and were subtracted from the raw signal. ΔA is the peak height relative to the baseline, $t_R = L/\bar{u}$ the retention time and D the Fick diffusion coefficient.

The two absorption curves in Fig. S2a show that the initial pulse of solute was stretched into a Gaussian shape as described by Eq. (1). The Fick diffusion coefficient was then extracted by fitting Eq. (1) to the IR detector signal. Fig. S2b illustrates exemplary experimental data together with the fitting curve after subtracting the baseline.

SIMULATION DETAILS

All molecular simulations were carried out with the program *ms*2[8] in a cubic volume with periodic boundary conditions where the cut-off radius was set to $r_c = 17.5$ Å. Lennard-Jones long range interactions were considered using angle averaging[9]. Electrostatic long-range corrections were considered by the reaction field technique with conducting boundary conditions ($\epsilon_{RF} = \infty$). Newton's equations of motion were solved with a fifth-order Gear predictor-corrector numerical integrator. The temperature was controlled by velocity scaling. In all simulations, the integration time step was 0.99 fs. The uncertainties of the predicted values were estimated with a block averaging method[10].

The chemical potential of each component was obtained with Widom's[11] particle insertion method from equilibrium molecular dynamics simulations in the isobaric-isothermal (NpT) ensemble. The simulations contained 2000 molecules and were equilibrated over 5×10^5 time steps, followed by a production run over 2×10^6. The density at the specified temperature, pressure and composition obtained from NpT simulations was employed for a subsequent simulation with 3000 molecules in the canonic (NVT) ensemble to calculate the transport properties. NVT simulations were equilibrated over 5×10^5 time steps with production runs of 2.5×10^7 time steps. The correlation functions were calculated using up to 2×10^5 independent time origins of the correlation functions with a sampling length of 40 ps for

all mixtures. This extensive length of the autocorrelation functions was chosen so that long-time tails corrections were not necessary. The separation between the time origins was chosen such that all correlation functions had decayed at least to 1/e of their normalized value to achieve their time independence[12]. The statistical uncertainties of the sampled data were estimated with the block averaging method by Flyvberg und Petersen[13]. In the case of derived quantities, the uncertainties were determined by the error propagation law.

The Yeh-Hummer[14] system size correction was applied to the predicted intra- and self-diffusion coefficients, which did not exceed 4% of the diffusivity values. For both mutual diffusion coefficients, size-corrections were considered and found to be within the statistical error.

The Maxwell-Stefan diffusion coefficient $Đ$ was obtained directly from the Onsager phenomenological coefficients[15] that are based on collective velocity correlation functions. It is straightforwardly related to the Fick diffusion coefficient D through the thermodynamic factor, which is given by

$$\Gamma = 1 + x_1 \left(\frac{\partial \ln \gamma_1}{\partial x_1} \right)_{T,p} = 1 + x_2 \left(\frac{\partial \ln \gamma_2}{\partial x_2} \right)_{T,p} , \tag{2}$$

where γ_i and x_i stand for the activity coefficient and mole fraction of component i. The Maxwell-Stefan diffusion coefficient can thus be transformed to the Fick diffusion coefficient and vice versa, if the thermodynamic factor is known. The mole fraction derivative of the activity coefficient γ_i and thus the thermodynamic factor was calculated from

$$\left. \frac{\partial \ln \gamma_i}{\partial x_j} \right|_{T,p,x_{k,k\neq j=1\ldots n-1}} = \left. \frac{\partial (\beta \tilde{\mu}_i - \ln x_i)}{\partial x_j} \right|_{T,p,x_{k,k\neq j=1\ldots n-1}} , \tag{3}$$

where $\beta = 1/(kT)$. Therein, μ_i is the chemical potential of component i and $\beta\tilde{\mu}_i(T,p,\mathbf{x})$ was directly sampled by molecular simulation. The right-hand side of Eq. (3) was approximated by numerical differentiation

$$\frac{\partial f(x_i)}{\partial x_i} \simeq \frac{f(x_{a+h}) - f(x_{a-h})}{2h}. \tag{4}$$

SELF-DIFFUSION COEFFICIENT OF PURE CO_2 AND CH_4

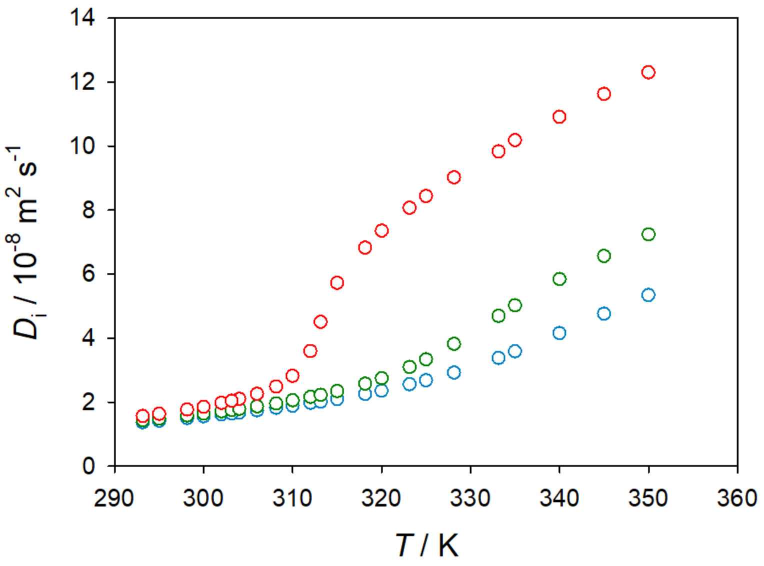

FIG. S3. Temperature dependence of the self-diffusion coefficient of CO_2 along the isobars p = 9 MPa (red), 12.5 MPa (green) and 14.7 MPa (blue) predicted by molecular simulation. The statistical uncertainties are within symbol size.

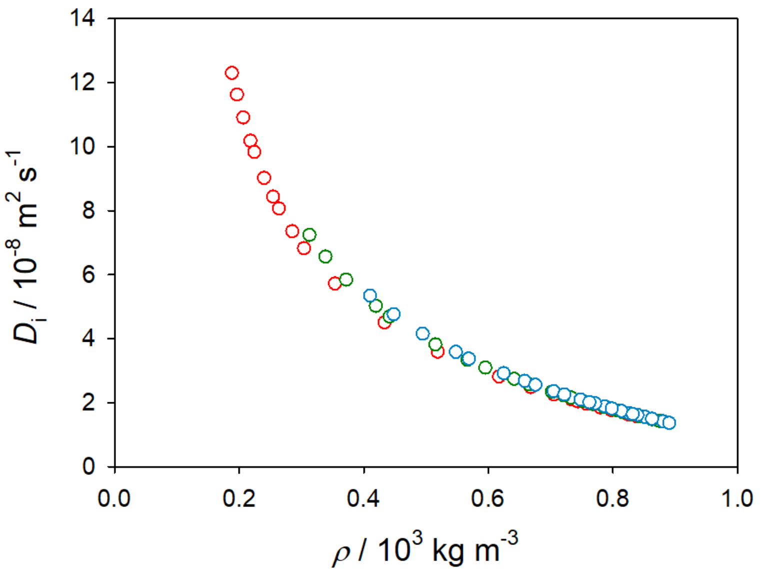

FIG. S4. Density dependence of the self-diffusion coefficient of CO_2 along the isobars p = 9 MPa (red), 12.5 MPa (green) and 14.7 MPa (blue) predicted by molecular simulation. The statistical uncertainties are within symbol size.

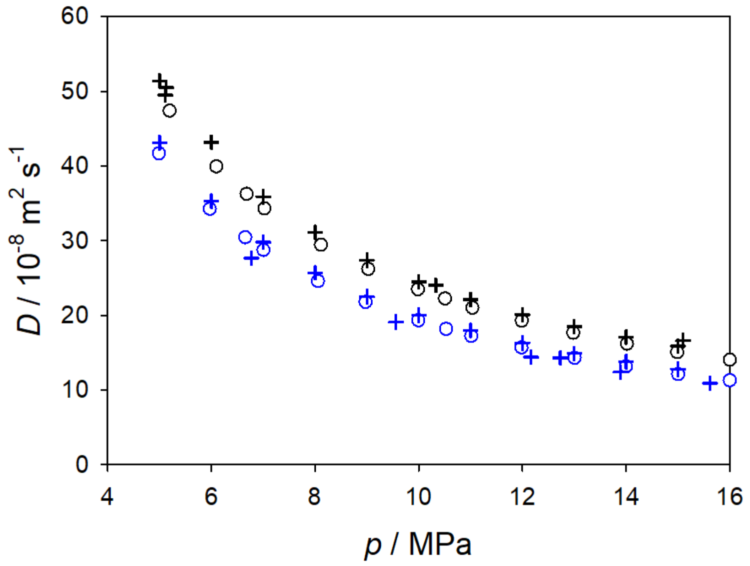

FIG. S5. Pressure dependence of the self-diffusion coefficient of CH_4 along the isotherms T = 298.15 K (blue) and 323.15 K (black). Simulation results (circles) are compared with experimental data[16,17](crosses). The statistical uncertainties are within symbol size.

SHEAR VISCOSITY OF PURE CO_2

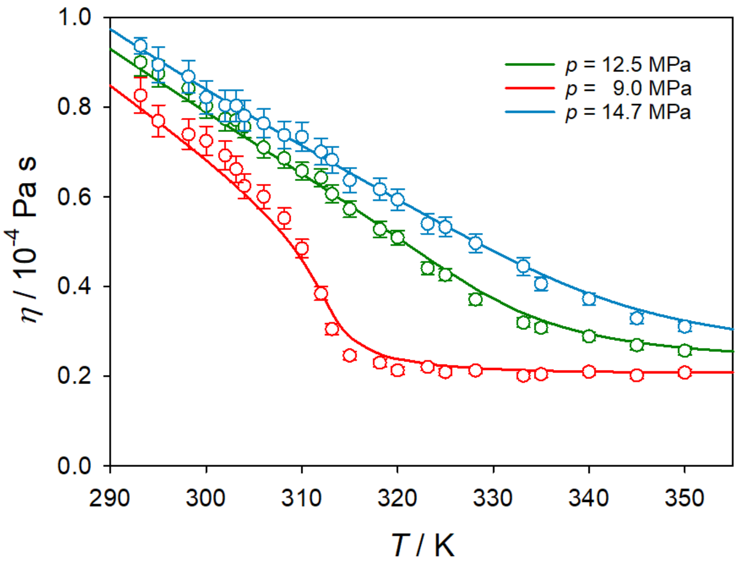

FIG. S6. Temperature dependence of the shear viscosity of CO_2 along the isobars $p = 9$ MPa (red), 12.5 MPa (green) and 14.7 MPa (blue). Simulation results (circles) are compared with the shear viscosity from the Laesecke and Muzny[18] correlation (lines).

THERMODYNAMIC FACTOR

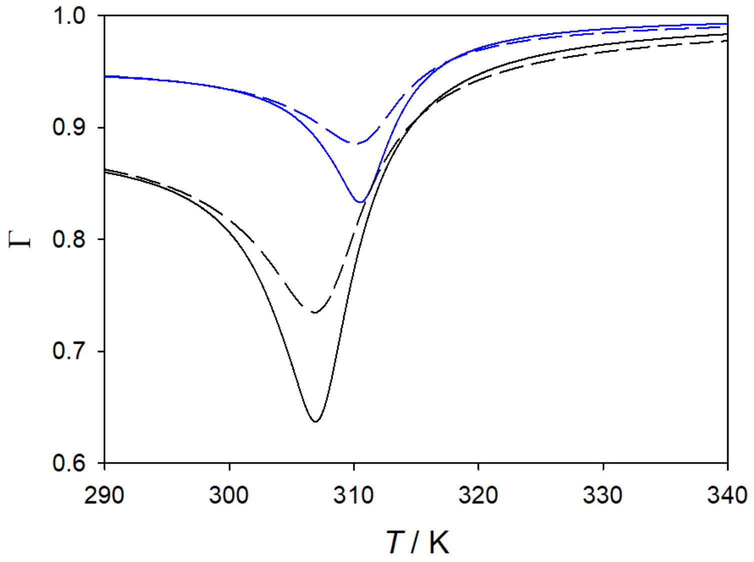

FIG. S7. Temperature dependence of the thermodynamic factor of the CH_4/CO_2 mixture with $x_{CH4} = 0.02$ mol mol^{-1} (blue) and 0.05 mol mol^{-1} (black) along the isobar $p = 9.0\,\mathrm{MPa}$ obtained from the Peng-Robinson[19](dashed lines) and GERG-2008[20] (solid lines) equations of state.

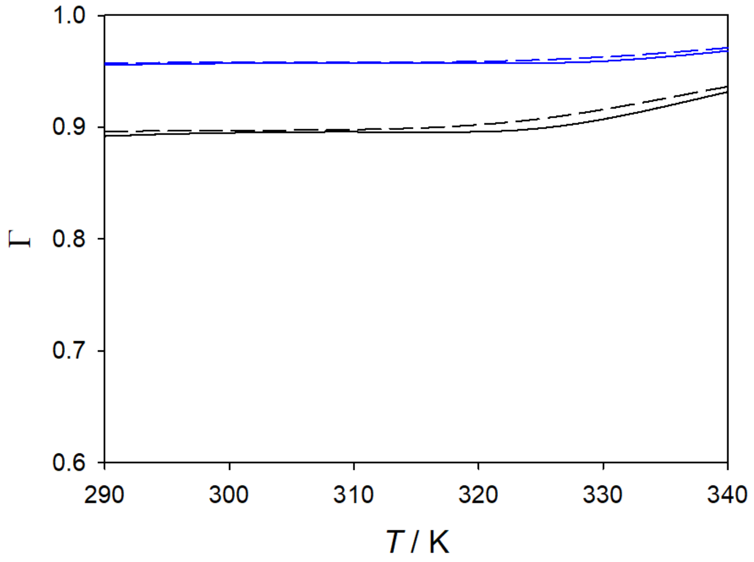

FIG. S8. Temperature dependence of the thermodynamic factor of the CH_4/CO_2 mixture with $x_{CH4} = 0.02$ mol mol^{-1} (blue) and 0.05 mol mol^{-1} (black) along the isobar $p = 14\,\mathrm{MPa}$ obtained from the Peng-Robinson[19] (dashed lines) and GERG-2008[20] (solid lines) equations of state.

NUMERICAL VALUES OF THE SIMULATION RESULTS

TABLE S1: Density ρ, Fick D, intradiffusion coefficients D_{CO2}, D_{CH4} and thermodynamic factor Γ of the CH_4/CO_2 mixture with $x_{CH4} = 0.01$ mol mol^{-1} along the isobars $p = 9.0$, 12.5 and 14.7 MPa predicted by molecular simulation. The numbers in parentheses denote the statistical uncertainty in the last digits.

$p = 9$ MPa					
T K	ρ mol L^{-1}	D 10^{-8} m^2 s^{-1}	D_{CO2} 10^{-8} m^2 s^{-1}	D_{CH4} 10^{-8} m^2 s^{-1}	Γ
293.15	18.77	2.33 (25)	1.62 (3)	2.19 (5)	0.971 (10)
295.00	18.44	2.30 (27)	1.68 (3)	2.25 (6)	0.972 (9)
298.15	17.79	2.55 (30)	1.83 (4)	2.42 (6)	0.965 (9)
300.00	17.37	2.14 (27)	1.92 (4)	2.69 (7)	0.960 (10)
302.00	16.85	2.81 (28)	2.04 (5)	2.94 (7)	0.962 (10)
303.15	16.48	2.97 (29)	2.14 (4)	3.00 (7)	0.966 (15)
304.00	16.18	2.68 (29)	2.20 (5)	3.14 (7)	0.958 (13)
306.00	15.34	2.93 (29)	2.41 (5)	3.38 (8)	0.957 (14)
308.15	14.51	3.18 (31)	2.66 (5)	3.83 (7)	0.933 (17)
310.00	12.45	4.66 (41)	3.31 (6)	4.94 (9)	0.915 (23)
312.00	9.89	5.65 (44)	4.45 (7)	6.82 (11)	0.885 (21)
313.15	8.58	7.63 (56)	5.03 (8)	8.32 (12)	0.937 (18)
315.00	7.58	9.45 (65)	6.10 (9)	9.44 (16)	0.961 (14)
318.15	6.70	10.46 (78)	7.07 (9)	10.93 (14)	0.977 (10)
320.00	6.30	12.11 (85)	7.60 (11)	11.76 (20)	0.984 (9)
323.15	5.85	12.74 (95)	8.29 (12)	12.85 (18)	0.989 (9)
325.00	5.67	13.65 (85)	8.60 (13)	13.46 (21)	0.991 (10)
328.15	5.37	13.81 (97)	9.21 (14)	14.46 (22)	0.992 (8)
333.15	5.02	14.28 (91)	10.04 (14)	15.57 (21)	0.994 (9)
$p = 12.5$ MPa					
T K	ρ mol L^{-1}	D 10^{-8} m^2 s^{-1}	D_{CO2} 10^{-8} m^2 s^{-1}	D_{CH4} 10^{-8} m^2 s^{-1}	Γ
293.15	19.68	1.84 (11)	1.47 (2)	1.99 (2)	0.976 (9)
295.00	19.42	1.92 (11)	1.53 (2)	2.05 (2)	0.975 (9)
298.15	18.94	2.26 (13)	1.62 (2)	2.19 (2)	0.968 (8)
300.00	18.65	2.37 (14)	1.68 (2)	2.28 (2)	0.965 (9)
302.00	18.32	2.51 (14)	1.76 (3)	2.41 (3)	0.970 (10)
303.15	18.12	2.36 (14)	1.80 (3)	2.44 (3)	0.967 (10)

304.00	17.97	2.38 (14)	1.84 (3)	2.50 (3)	0.968 (9)
306.00	17.61	2.63 (16)	1.92 (3)	2.65 (3)	0.971 (10)
308.15	17.19	2.59 (16)	2.02 (3)	2.85 (3)	0.967 (10)
310.00	16.80	2.87 (16)	2.12 (3)	2.87 (3)	0.966 (11)
312.00	16.38	2.88 (18)	2.24 (3)	3.05 (3)	0.960 (10)
313.15	16.10	2.95 (18)	2.31 (3)	3.21 (3)	0.961 (11)
315.00	15.64	3.43 (20)	2.43 (4)	3.40 (4)	0.961 (12)
318.15	14.76	3.66 (22)	2.70 (4)	3.78 (4)	0.959 (13)
320.00	14.18	3.80 (23)	2.88 (4)	3.96 (4)	0.958 (13)
323.15	13.12	4.67 (27)	3.24 (4)	4.60 (5)	0.957 (14)
325.00	12.45	4.78 (26)	3.50 (5)	5.03 (5)	0.964 (14)
328.15	11.31	5.72 (33)	4.01 (5)	5.77 (6)	0.966 (13)
333.15	9.75	6.73 (37)	4.86 (7)	7.08 (7)	0.972 (13)

$p = 14.7$ MPa

T K	ρ mol L^{-1}	D 10^{-8} m^2 s^{-1}	D_{CO2} 10^{-8} m^2 s^{-1}	D_{CH4} 10^{-8} m^2 s^{-1}	Γ
293.15	20.06	1.94 (28)	1.40 (4)	1.92 (6)	0.971 (10)
295.00	19.86	1.93 (27)	1.44 (4)	1.97 (6)	0.972 (9)
298.15	19.42	2.12 (23)	1.53 (4)	2.08 (6)	0.970 (10)
300.00	19.17	2.26 (26)	1.58 (4)	2.14 (6)	0.976 (10)
302.00	18.92	2.28 (24)	1.63 (4)	2.18 (6)	0.969 (10)
303.15	18.72	2.39 (24)	1.69 (5)	2.25 (6)	0.966 (10)
304.00	18.58	2.28 (26)	1.71 (4)	2.28 (6)	0.968 (12)
306.00	18.32	2.40 (25)	1.76 (4)	2.35 (6)	0.979 (14)
308.15	17.93	2.53 (26)	1.86 (5)	2.52 (7)	0.969 (16)
310.00	17.60	2.60 (28)	1.94 (5)	2.63 (7)	0.964 (20)
312.00	17.30	2.70 (29)	2.00 (4)	2.70 (7)	0.987 (21)
313.15	17.11	2.82 (31)	2.01 (5)	2.83 (7)	0.964 (18)
315.00	16.74	2.92 (31)	2.15 (5)	2.92 (7)	0.967 (15)
318.15	16.13	3.21 (32)	2.33 (6)	3.20 (8)	0.976 (10)
320.00	15.83	3.22 (33)	2.41 (5)	3.30 (7)	0.975 (9)
323.15	15.03	3.59 (37)	2.67 (6)	3.67 (8)	0.978 (9)
325.00	14.69	3.87 (36)	2.75 (5)	3.84 (7)	0.982 (11)
328.15	13.92	4.27 (38)	3.01 (6)	4.29 (8)	0.985 (11)
333.15	12.17	4.72 (41)	3.68 (7)	5.35 (9)	0.984 (14)

REFERENCES

[1]NIST, *NIST Chemistry Webbook , SRD9* (NIST, 2011), http://webbook.nist.gov

[2]J.M.H. Levelt Sengers, U.K. Deiters, U. Klask, G. Swidersky, M. Schneider, Int. J. Thermophys. **14**, 893–922 (1993)

[3]L. Janssen, Chem. Eng. Sci. **31**, 215–218 (1976)

[4]A. Alizadeh, C.A. Nieto de Castro, W.A. Wakeham, Int. J. Thermophys. **1**, 243–284 (1980)

[5]A. Akgerman, C. Erkey, M. Orejuela, Ind. Eng. Chem. Res. **35**, 911–917 (1996)

[6]S. Ancherbak, C. Santos, J. Legros, A. Mialdun, V. Shevtsova, Eur. Phys. J. E **39**, 111 (2016)

[7]V. Sechenyh, J.C. Legros, V. Shevtsova, C. R. Mecanique **341**, 490–496 (2013)

[8]G. Rutkai, A. Köster, G. Guevara-Carrion, T. Janzen, M. Schappals, C.W. Glass, M. Bernreuther, A. Wafai, S. Stephan, M. Kohns et al., Comp. Phys. Commun. **221**, 343–351 (2017)

[9]R. Lustig, Mol. Phys. **65**, 175–179 (1988)

[10]M.P. Allen, D.J. Tildesley, *Computer Simulation of Liquids* (Clarendon Press, Oxford, 1987)

[11]B. Widom, J. Chem. Phys. **39**, 2808–2812 (1963)

[12]M. Schoen, C. Hoheisel, Mol. Phys. **52**, 33–56 (1984)

[13]H. Flyvbjerg, H.G. Petersen, J. Chem. Phys. **91**, 461–466 (1989)

[14]I.C. Yeh, G. Hummer, J. Phys. Chem. B **108**, 15873–15879 (2004)

[15]R. Krishna, J.A. Wesselingh, Chem. Eng. Sci. **52**, 861–911 (1997)

[16]S. Takahashi, H. Iwasaki, Rev. Phys. Chem. Jpn. **46**, 88–94 (1976)

[17]D.E. Woessner, B.S. Snowden, R.A. George, J.C. Melrose, Ind. Eng. Chem. Fundam. **8**, 779–787 (1969)

[18]A. Laesecke, C.D. Muzny, J. Phys. Chem. Ref. Data **46**, 013107 (2017)

[19]D.Y. Peng, D.B. Robinson, Ind. Eng. Chem. Fundam. **15**, 59–64 (1976)

[20]O. Kunz, W. Wagner, J. Chem. Eng. Data **57**, 3032–3091 (2012)
